# Non-differential measurement error does not always bias diagnostic likelihood ratios towards the null

**DOI:** 10.1186/1742-7622-3-7

**Published:** 2006-07-17

**Authors:** GT Fosgate

**Affiliations:** 1 Department of Veterinary Integrative Biosciences, College of Veterinary Medicine and Biomedical Sciences, Texas A&M University, College Station, TX, 77843-4458, USA

## Abstract

Diagnostic test evaluations are susceptible to random and systematic error. Simulated non-differential random error for six different error distributions was evaluated for its effect on measures of diagnostic accuracy for a brucellosis competitive ELISA. Test results were divided into four categories: <0.25, 0.25 – 0.349, 0.35 – 0.499, and ≥ 0.50 proportions inhibition for calculation of likelihood ratios and diagnostic odds ratios. Larger variance components of the error structure resulted in larger accuracy attenuations as measured by the area under the receiver-operating characteristic curve and systematic components appeared to cause little bias. Added error caused point estimates of likelihood ratios to be biased towards the null value (1.0) for all categories except 0.25 – 0.349. Results for the 0.35 – 0.499 category also extended beyond the null value for some error structures. Diagnostic odds ratios were consistently biased towards the null when the <0.25 category was considered the reference level. Non-differential measurement error can lead to biased results in the quantitative evaluation of ELISA and the direction is not always towards the null value.

## Background

The goal of epidemiologic investigations is the collection of valid data leading to a precise estimate of a population parameter (e.g. measure of association). For the purpose of this discussion, an estimate of a parameter will be considered biased if the expected value (over indefinite replications) is not the true value [1,2]. A study or process is considered biased if a systematic error is present in study design, data collection, or data analysis [2,3]. Systematic error, using a slight modification of a standard dictionary definition [4], can be defined as a persistent error having a nonzero mean that cannot be attributed entirely to chance but to inaccuracy inherent in the system of measurement. A random error develops from imprecision in a measuring instrument or protocol used to collect data for study. A random error in absence of systematic error will not result in bias if on average the measured value is still the true population value. The effect of random errors will be reduced by increasing sample size or number of measurements taken from each sampling unit. Systematic error will not be reduced by increasing sample size because it does not result from imprecise measurements.

Epidemiologic investigations must consider the potential effects of both systematic and random errors on study results. The odds ratio (OR) is frequently the measure of association estimated in studies concerning etiology and the likelihood ratio (LR) is commonly estimated for evaluation of diagnostic tests. Odds ratios for diagnostic purposes can also be estimated that quantify the change in the odds of infection (or disease) resulting from a positive test result [5].

Estimates of LRs and diagnostic ORs can be affected by random and systematic errors similar to other epidemiologic measures of association. The error in detection of the analyte (biologic substance measured by a diagnostic assay) must exert its effect through misclassification of the test result. The ability of the analyte to predict infection (or lack of infection) in an individual determines its usefulness or accuracy for diagnosis. Accuracy of a testing system is measured by its sensitivity (probability of correctly classifying infected individuals) and specificity (probability of correctly classifying uninfected individuals). Accuracy can be measured at a single or over multiple positive cutoff values.

The evaluation of tests over multiple cutoffs can be performed through estimation of LRs or performing receiver-operating characteristic (ROC) analyses. The LR is a measure of association that quantifies how many more times likely a test result is from an infected individual compared to one that is uninfected. It is calculated as the ratio of the probability that an infected individual will have that test result to the probability that an uninfected individual would have that same result [6]. Calculation of LRs for tests with quantitative outcomes (e.g. titers, optical densities) necessitates dividing up the possible range of test results into categories. Likelihood ratios are also mathematically related to ROC curves as the slope between adjacent test result categories [7].

Receiver-operating characteristic (ROC) curves are formed by plotting 1 – specificity (x-axis) by sensitivity (y-axis) over multiple positive cutoff values [8]. The area under a ROC curve (AUC) is defined as the probability that a randomly selected infected individual will have a greater test result than a randomly selected uninfected individual, and is considered a measure of overall discriminating ability of the test [9]. The precision of a diagnostic testing system will affect the overall accuracy and is often measured as the coefficient of variation (CV), which is calculated as the standard deviation of measurements on the same sample divided by the mean of the measurements. The CV quantifies the random measurement error inherent in the diagnostic system.

Measurement error associated with the analyte could theoretically be differential or non-differential. Differential measurement error is defined as an error whose magnitude or direction is different for individuals who have the outcome (eg. infection) compared to those without the outcome. Non-differential measurement error is an error that is independent of outcome status; the direction and magnitude is equal for those with and without the outcome. Measurement error can lead to misclassification that is differential or non-differential. The effect of measurement error and misclassification on LRs could not be found in the currently available peer-reviewed literature. The direction of bias in estimates of ORs and risk ratios with differential misclassification cannot be predicted [10-12], however, non-differential misclassification of an exposure has been shown to result in measures of association to be consistently biased towards the null when evaluated in a 2 × 2 table [1,10-14] except in unrealistically extreme situations [1,10,15]. When the exposure is classified into more than two categories (higher-order tables) the direction of deviation is no longer consistently towards the null value with non-differential misclassification [10,15-18]. However, even in situations with more than two levels of exposure, the measures of association will be biased towards the null when calculated for the categories involved in the misclassified exposure [19,20].

A quantitative exposure that is categorized into three levels will often assign the lowest category (level 1) as the reference level. The usual ORs evaluating the effect of exposure are calculated comparing level 2 to level 1 and level 3 to level 1. If non-differential misclassification occurs only between exposure levels 2 and 3, for instance, then the usual ORs could be biased towards or away from the null value, however, the OR calculated between level 2 and level 3 (not usually reported) would consistently be biased towards the null. The exception to this rule is when misclassification is so extreme that the probability of incorrect classification is more likely than correct classification [19,20].

The effects of misclassification on measures of association are often studied by creating hypothetical data distributions, but simulation studies using actual data can also be employed [21,22]. Simulation studies have the advantage of defining probability distributions rather than creating extreme and potentially unrealistic situations. The objective of this study was to investigate the effects of non-differential measurement error on AUC, LRs, and diagnostic ORs calculated for a test categorized into four levels using real data and simulated error structures.

## Analysis

### Data source

Brucellosis is a major disease problem worldwide [23] associated with chronic debilitating infections in people and reproductive failure in domestic animals. *Brucella *species that cause disease in people include *B. abortus *(primary reservoir is cattle and water buffalo), *B. melitensis *(sheep and goats), and *B. suis *(swine) [24]. Cattle and domestic water buffalo in Trinidad have been found to be infected with *B. abortus *[25] and the data used for this simulation study are results from a brucellosis competitive ELISA (c-ELISA) in 391 cattle and 381 domestic water buffalo (*Bubalus bubalis*) of Trinidad. Evaluation of this assay has been reported elsewhere [26] and results from both species were pooled in a single analysis for purpose of these evaluations. The brucellosis status (infected or uninfected with *B. abortus*) was determined using results from multiple diagnostic tests in a no gold standard analysis. The most likely infection status based on this analysis was assumed the true status. This classification resulted in 126 cattle and water buffalo infected with *B. abortus *and 656 uninfected cattle and water buffalo.

The c-ELISA is a quantitative test where sample results are reported as the proportion of inhibition compared to a conjugate-only control (no serum added). Each test and control sample had optical density (OD) values measured in duplicate and the formula to calculate the proportion inhibition (PI) is included below.



The theoretical limits are therefore zero to one with values closer to one being more positive (higher level of competing antibodies). Negative values occur infrequently when the OD of the sample is greater than the conjugate control.

### Data simulation

The data measured when performing an ELISA is the degree of color change, or OD, that quantifies the amount of antibodies in the serum. The observed mean OD values for test sera and conjugate-only controls from each ELISA plate were assumed to represent the true biologic value for purpose of these simulations. Commercially available software [27] was used to incorporate error distributions to both sample and control mean values independently. After addition of error to original mean OD values, the PI was re-calculated for each sample.

Conjugate-only control samples contain no competing antibodies and therefore the color change (i.e. OD) should be equal to a baseline level. Variation in the measured values for these controls represents the random error associated with the assay. Therefore, mean ODs measured for the duplicate conjugate controls on the original ELISA plates were used to estimate the inherent error of the testing system and determine simulation error distributions. Normal distributions with means of 0, 0.1, -0.1 and standard deviation of 0.12 and mean of 0 and standard deviation of 0.24 were evaluated as part of the study. A value of 0.1 was chosen for a mean because it was the interquartile range for the average of duplicate conjugate control values on each ELISA plate. A standard deviation of 0.12 was chosen because this was the standard deviation of all original mean conjugate control values. A random sample from these distributions was added to observed mean OD values. Lognormal distributions were used to add an error structure that varied depending upon observed ODs. The scale (μ) parameter of these distributions was calculated as the observed mean OD of the particular sample divided by the mean OD of all sample values. The shape (σ) parameters investigated were 0.12 and 0.24. A random selection from these distributions was multiplied by the observed mean OD to calculate simulated values. Simulated mean ODs were not truncated in range and calculated PI values could be less than zero and greater than one.

Test results were divided into four categories: <0.25, 0.25 – 0.349, 0.35 – 0.499, and ≥ 0.50 PI. These categories were based on an evaluation of this assay [28] with the original six categories collapsed to four to reduce complexity of simulations and increase the number of infected and uninfected individuals in the lower most and upper most categories, respectively. Category-specific LRs [7] were calculated for each of the four categories as the proportion of infected individuals in each category divided by the proportion of uninfected individuals within that same category. Diagnostic ORs were calculated comparing the three higher test result categories to the lowest category as the baseline, or reference level. Sensitivity and specificity were calculated for the c-ELISA at all possible cutoff values from 0.01 to 0.99 PI at 0.01 intervals. Area under the ROC curve was calculated as an overall measure of diagnostic accuracy using the trapezoid method [29]. The average sensitivity between adjacent cutoffs was the mean height of the trapezoid and base width was the difference in adjacent specificities.

Six simulation studies were performed independently assessing the impact of added error distributions to the original observed data. Monte Carlo sampling was performed of these error distributions independently for each test sample and conjugate control over 10,000 iterations. Error was added to all mean OD values at each iteration, new PIs were calculated, and diagnostic accuracy measures (AUC, LR, OR) were determined. The mean, median, standard deviation, minimum, and maximum values of PIs for infected and uninfected individuals were calculated at each iteration. Median values and percentiles over these 10,000 iterations were used as point estimates and confidence intervals, respectively for descriptive statistics and all investigated AUCs, LRs, and ORs.

### Simulation results

**Figure 1:**
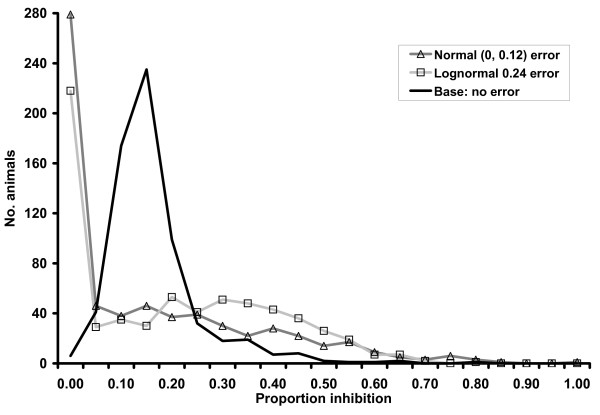
Distribution of c-ELISA proportion inhibition results for 656 *B. abortus *uninfected cattle and water buffalo from Trinidad with added error from a single iteration of a simulation study and summed over 5% proportion inhibition intervals.

**Figure 2:**
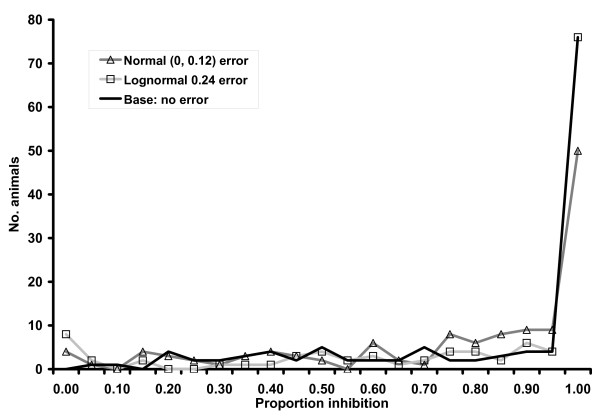
Distribution of c-ELISA proportion inhibition results for 126 *B. abortus *infected cattle and water buffalo with added error from a single iteration of a simulation study and summed over 5% proportion inhibition intervals.

The six added error structures caused mean PI values to have greater range and larger standard deviations for both infected and uninfected groups of individuals compared to original values and decreased overall test accuracy as measured by AUC (Table 1). Distribution of c-ELISA PI values for Normal (0, 0.12) and lognormal (0.24) error structures were noticeably different from the original distribution for uninfected individuals (Fig 1) and relatively similar for infected individuals (Fig 2). Added error with different means but the same standard deviations resulted in visually similar distributions (data not shown). Distribution of PIs in uninfected individuals peaked at zero because all lower extreme values were included in the 0–5% test result category. In general, distribution of PIs with added error had a wider (less precise) distribution, which resulted in more overlap with distribution of infected individuals and lowered overall test accuracy. Added error caused point estimates of LRs to be biased towards the null value (1.0) for all categories except 0.25 – 0.349 (Table 2). Results for the 0.35 – 0.499 category also extended beyond the null value for some error structures. Diagnostic ORs calculated with the lowest category as baseline were consistently biased towards the null for all evaluated error structures. Error structures with larger variance resulted in more bias for both LRs and ORs.

**Table 1:** Descriptive statistics for proportion inhibition (PI) and area under the receiver-operating characteristic curve (AUC) for a brucellosis c-ELISA used in 126 infected and 656 uninfected cattle and water buffalo of Trinidad incorporating multiple error structures. Median values for each statistic (eg. mean) are reported over 10,000 iterations of the Monte Carlo simulation.

Error structure	Infected		Uninfected		AUC (90% Interval)*
		
	Mean (sd)	Minimum, median, maximum	Mean (sd)	Minimum, median, maximum	
None (standard)	0.812 (0.273)	0.050, 0.983, 1.006	0.137 (0.091)	-0.033, 0.119, 0.752	0.973
Normal (0, 0.12)	0.810 (0.291)	-0.033, 0.921, 1.210	0.130 (0.150)	-0.328, 0.129, 0.768	0.959 (0.945, 0.972)
Lognormal 0.12	0.809 (0.285)	-0.136, 0.983, 1.006	0.118 (0.207)	-0.685, 0.137, 0.752	0.952 (0.934, 0.968)
Normal (0.1, 0.12)	0.753 (0.271)	-0.031, 0.856, 1.123	0.122 (0.139)	-0.301, 0.120, 0.714	0.959 (0.944, 0.972)
Normal (-0.1, 0.12)	0.877 (0.315)	-0.036, 0.996, 1.316	0.140 (0.162)	-0.361, 0.139, 0.829	0.959 (0.945, 0.972)
Normal (0, 0.24)	0.806 (0.344)	-0.273, 0.880, 1.459	0.108 (0.266)	-0.906, 0.133, 0.877	0.933 (0.907, 0.953)
Lognormal 0.24	0.800 (0.320)	-0.542, 0.982, 1.006	0.059 (0.419)	-2.074, 0.143, 0.785	0.928 (0.899, 0.950)

*Interval formed as the 5^th ^and 95^th ^percentiles of Monte Carlo simulations

sd = standard deviation

**Table 2:** Results of adding six measurement error structures on the likelihood ratio (LR) and diagnostic odds ratio (OR) for a brucellosis c-ELISA used in 126 infected and 656 uninfected cattle and water buffalo of Trinidad over four categories of proportion inhibition (PI). Results calculated over 10,000 iterations of the Monte Carlo simulation.

Error structure	Result category (PI)	No. infected*	No. uninfected*	LR* (90% Interval^†^)	LR difference*	OR* (90% Interval^†^)	OR difference*
None	<0.25	8	586	0.070	NA	1.0	NA
(standard)	0.25 – 0.349	5	38	0.675	NA	9.64	NA
	0.35 – 0.499	11	17	3.32	NA	47.4	NA
	≥0.50	102	5	105	NA	1494	NA

Normal	<0.25	8	522	0.083 (0.051, 0.116)	0.013	1.0 (referent)	NA
(0, 0.12)	0.25 – 0.349	5	80	0.337 (0.136, 0.630)	-0.337	4.14 (1.36, 9.90)	-5.50
	0.35 – 0.499	9	34	1.33 (0.72, 2.32)	-1.99	16.35 (7.37, 35.8)	-31.0
	≥0.50	103	9	59.2 (40.2, 105)	-45.3	749 (431, 1415)	-745

Lognormal	<0.25	8	469	0.089 (0.049, 0.133)	0.019	1.0 (referent)	NA
0.12	0.25 – 0.349	5	100	0.244 (0.088, 0.485)	-0.430	2.79 (0.78, 7.57)	-6.85
	0.35 – 0.499	9	64	0.707 (0.338, 1.42)	-2.61	8.13 (3.10, 21.5)	-39.3
	≥0.50	104	11	49.4 (23.8, 104)	-55.2	582 (239, 1288)	-913

Normal	<0.25	9	543	0.088 (0.057, 0.122)	0.018	1.0 (referent)	NA
(0.1, 0.12)	0.25 – 0.349	6	71	0.427 (0.181, 0.801)	-0.247	4.89 (1.69, 11.7)	-4.74
	0.35 – 0.499	10	26	1.83 (0.98, 3.24)	-1.49	21.2 (9.70, 44.6)	-26.2
	≥0.50	101	6	86.3 (53.3, 171)	-18.3	994 (580, 1980)	-501

Normal	<0.25	7	499	0.078 (0.046, 0.113)	0.008	1.0 (referent)	NA
(-0.1, 0.12)	0.25 – 0.349	5	90	0.264 (0.101, 0.506)	-0.410	3.44 (1.03, 8.89)	-6.20
	0.35 – 0.499	8	43	0.954 (0.488, 1.66)	-2.36	12.5 (5.25, 28.2)	-34.9
	≥0.50	105	13	41.8 (28.6, 66.7)	-62.8	555 (321, 1065)	-939

Normal	<0.25	10	444	0.120 (0.068, 0.176)	0.049	1.0 (referent)	NA
(0, 0.24)	0.25 – 0.349	4	89	0.254 (0.089, 0.496)	-0.421	2.14 (0.61, 5.56)	-7.50
	0.35 – 0.499	8	79	0.540 (0.267, 0.966)	-2.78	4.59 (1.85, 10.8)	-42.8
	≥0.50	103	34	15.7 (10.7, 24.7)	-88.9	135 (73.0, 270)	-1359

Lognormal	<0.25	10	401	0.124 (0.065, 0.192)	0.054	1.0 (referent)	NA
0.24	0.25 – 0.349	3	75	0.237 (0.062, 0.500)	-0.438	1.91 (0.43, 5.44)	-7.72
	0.35 – 0.499	7	99	0.366 (0.169, 0.675)	-2.95	3.00 (1.11, 7.62)	-44.4
	≥0.50	105	68	7.81 (4.68, 16.1)	-96.8	67.0 (30.1, 167)	-1427

*Median value of Monte Carlo simulations. †Interval formed as the 5^th ^and 95^th ^percentiles of Monte Carlo simulations.

NA = not applicable.

## Conclusion

The effect of non-differential random error in exposure measurement has been discussed in previous publications [21,30-32], and leads to measures of association being biased towards the null value except in unrealistically extreme situations. Overall accuracy of a quantitative diagnostic test, measured via the AUC, has been shown here to also be decreased (biased towards null value of 0.5) through addition of non-differential measurement error. The variance component of the measurement error structure appears to have an important effect on decreasing AUC and the systematic (mean) component of the error structure has little or no bearing on results when applied equally to all samples (ie. non-differential). This attenuation in accuracy is due to added variability spreading out the distribution of test results and creating more overlap between results from infected and uninfected individuals as shown in the figures. Values were not truncated during simulations despite the fact that biologically unusual values were observed as evidenced by the PI ranges. These observations did not unduly influence the analysis because they were considered equivalent to the boundary categories.

Likelihood ratios are derived from the odds version of Bayes' theorem [28], correspond to the added information provided by a test, and are used to update the prior odds of infection. The null value of a LR is one, which would correspond to a particular test result being equally likely in infected and uninfected individuals (would not affect prior probabilities). A previous study [31] demonstrated that non-differential, random measurement error in exposure determination without a systematic component, consistently led to attenuation in effect measures such as the OR. Results for diagnostic ORs agreed with this previous finding; however, LRs estimated in the present study were not consistently biased towards the null value. For example, the baseline (without error) LR for the 0.25 – 0.349 category was 0.675 and all evaluated error structures resulted in this LR (based on median simulated value) to be biased further away from one. The baseline LR for the 0.35 – 0.499 category was 3.32 and four of the evaluated error structures resulted in this measure to be biased to such an extent that the point estimates extended below the null value of one. Estimates of LRs and ORs were also mildly affected by the systematic component (mean) of the error structure. Unlike the AUC, these measures are dependent upon the underlying distribution of values because they are calculated for a small number of fixed categories.

The direction of bias is not easily described as being away from or towards the null value for investigated LRs. However, all LRs estimated from evaluated error structures could be described as being biased in a manner resulting in the test having less discriminating ability for its usual function at that category. For instance, the lowest category is often useful for "ruling out" infection given that (i.e. "negative") test result. The observed bias towards one causes a test result falling within this category to be less useful for that purpose. Larger test result categories are more positive (0.30 is the usual positive cutoff for the brucellosis c-ELISA [33]) and therefore a higher post-test probability of infection (compared to pre-test probability) would be the desired effect. All error structures resulted in LRs for these categories to be biased towards zero, which means that the test is less useful for this purpose (calculated post-test probability of infection lower than the true value). The observed direction of the biases in this study might have resulted from the underlying distributions of test results for infected and uninfected individuals and a different dataset might not demonstrate the same relationships.

A strength of the employed simulation procedure is that error distributions were added to the mean ODs measured from duplicate test and control samples. Optical densities, and not PIs, would be the values actually affected by measurement error. A similar analysis adding error to the PIs would not directly simulate this type of error. Lognormal error distributions were evaluated to simulate measurement error that depended on the magnitude of the measured value. In the example of a c-ELISA, higher OD values correspond to more negative (fewer competing antibodies) samples. Therefore, this error structure has a greater impact on the distribution of values in uninfected individuals as was seen in the presented figures. Investigated error structures might overestimate true measurement error and only a limited number of distributions were evaluated leading to difficulty in generalizing results to all possible error situations. However, added error distributions were based on true observations from the mean conjugate-only controls that have no competing antibodies. Therefore, variability inherent in these measurements should be a valid representation of the true variability of the testing system. It is expected that some sources of error would be dependent upon plate-level and day-level factors such as reagents, laboratory temperature, and incubation times that would be equal for both test and control samples. Therefore, the addition of non-differential error independently to test and control values represents the upper limit of possible effects on test accuracy measures.

Non-differential random error added via a probability distribution might result in differential misclassification of test result categories as evidenced in data presented in Table 2. The proportion of individuals misclassified in the four test result categories does not appear to be equal between infected and uninfected individuals. A similar finding has been reported for non-differential measurement error of exposure [21,31,32]. In this study, however, it is impossible to know which particular individuals were misclassified because only total counts could be calculated and an accurate assessment of the magnitude of misclassification could not be determined. It is only possible to know the net result of the misclassification and not the number of individuals incorrectly entering or leaving each category. The misclassification across the test result categories also depends upon the underlying distribution of values.

The true infection status of individuals in the evaluated dataset was not known and classification of individuals was performed based on results of a no gold standard test evaluation study. Therefore, the original data is expected to contain some results that were misclassified based on infection status. These errors are not expected to unduly affect results of the simulation study because they would apply equally to the baseline and error-augmented situations. The underlying distributions of test results in infected and uninfected individuals, however, might not adequately reflect the true distributions because of this potential misclassification.

This study shows that non-differential measurement error can lead to biased results in the evaluation of diagnostic tests with quantitative outcomes. It is especially important to recognize that LRs are not consistently biased towards the null even when measurement error is exclusively non-differential. These biases will not be reduced by simply increasing the sample size; it would be necessary to increase the number of observations on each sampling unit to reduce the impact of this error. It is therefore possible for an unbiased study (presence of random error without a systematic component) to yield biased population values through non-differential measurement error. This situation is possible when the population parameter to be estimated by the study (e.g. LR of a test) is not a simple one to one transformation of the data affected by measurement error (e.g. OD). The observed attenuation in AUC would be expected to occur in all situations involving non-differential measurement error, but the direction of bias in measured LRs would be expected to vary depending upon the amount of error and underlying distribution of test results.

## Abbreviations

OR – odds ratio

LR – likelihood ratio

ROC – receiver-operating characteristic

AUC – area under receiver-operating characteristic curve

CV – coefficient of variation

c-ELISA – competitive enzyme-linked immunosorbent assay

OD – optical density

PI – proportion inhibition

## Competing interests

The author declares that he has no competing interests.

## Authors' contributions

GTF performed all analyses and wrote the manuscript without substantive contributions from other investigators.
